# SARS-CoV-2 antibody response after BBIBP-CorV (Sinopharm) vaccination in cancer patients: A case-control study

**DOI:** 10.3389/fmed.2022.1095194

**Published:** 2023-01-10

**Authors:** Fahimeh Safarnezhad Tameshkel, Shabnam Abedin Dargoush, Bahareh Amirkalali, Saeedeh Javadi, Ali Ghiaseddin, Yousef Alimohamadi, Ali Basi, Mahin Jamshidi Makiani, Farhad Zamani, Mohammad Hadi Karbalaie Niya

**Affiliations:** ^1^Gastrointestinal and Liver Diseases Research Center, Iran University of Medical Sciences, Tehran, Iran; ^2^Stem Cell and Regenerative Medicine Center of Excellence, Tehran University of Medical Sciences, Tehran, Iran; ^3^Department of Internal Medicine, School of Medicine, Firoozgar Hospital, Iran University of Medical Sciences, Tehran, Iran; ^4^Department of Chemistry, Michigan State University, East Lansing, MI, United States; ^5^Health Research Center, Life Style Institute, Baqiyatallah University of Medical Sciences, Tehran, Iran; ^6^Department of Hematology and Oncology, School of Medicine, Firoozgar Hospital, Iran University of Medical Sciences, Tehran, Iran; ^7^Antimicrobial Resistant Research Center, Iran University of Medical Sciences, Tehran, Iran; ^8^Department of Infectious Disease, School of Medicine, Firoozgar General Hospital, Iran University of Medical Sciences, Tehran, Iran; ^9^Department of Virology, School of Medicine, Iran University of Medical Sciences, Tehran, Iran

**Keywords:** Sinopharm (BBIBP-CorV), cancer, COVID-19 vaccine, anti-sRBD antibody response, seroconversion, anti-N IgG, SARS-CoV-2

## Abstract

**Background:**

Long-term safety and efficacy of BBIBP-CorV vaccine especially in individuals with chronic diseases, like cancer, is under investigation. In the present prospective study, we aimed to evaluate severe acute respiratory syndrome-coronavirus-2 (SARS-CoV-2) antibody response with BBIBP-CorV vaccine in Iranian cancer patients.

**Methods:**

All the patients registered to receive BBIBP-CorV (Sinopharm) vaccine were divided into two groups of with (cases = 107) and without (controls = 45) history of cancer. Serum levels of SARS-CoV anti-spike recombinant receptor binding domain (anti-sRBD) and anti-nucleocapsid (anti-N) IgG serum levels were measured on days 0 (phase 0), 28–32 (phase I), and 56–64 (phase II) of vaccination. The data were analyzed using SPSS, version 22.

**Results:**

Totally, 152 individuals (67.1% females) with the mean age of 46.71 ± 15.36 years were included. Solid cancers included 87.8% of the cancer cases (46.7% gynecological and 31.8% gastrointestinal cancer). At Phases I and II, positive anti-sRBD IgG and anti-N IgG were significantly lower among the cases in total analysis. Side effects were not significantly different between the cases and controls. The lowest positive anti-sRBD IgG test was observed among the cancer patients who were simultaneously receiving chemotherapy (35.3%). Anti-sRBD IgG and anti-N IgG serum levels significantly increased at phases I and II in total analysis and in each group. In addition, serum anti-sRBD IgG increased during the three phases and it was significantly higher in the control group.

**Conclusion:**

Full vaccination of COVID-19 by BBIBP-CorV in immunocompromised patients such as cancer patients is safe and effective and could induce antibody response but in lower levels compared to healthy people. Probable causes to have minor antibody response found in males, older ages, individuals with BMI ≥ 25, those without past history of COVID-19 and with hematologic cancers. No significant side effects after vaccination were seen.

## Introduction

Severe acute respiratory syndrome-coronavirus-2 (SARS-CoV-2) causes infectious disease of COVID-19 in humans with clinical manifestations, such as cytokine storm, severe acute respiratory distress, pneumonia, and lymphopenia. Since December 2019 when it was first detected in the city of Wuhan, China, this virus has spread rapidly worldwide, affecting more than 220 million individuals and resulting in more than 4 million deaths. To fight against this pandemic, a variety of vaccines have been developed. Among the most widely used and WHO approved vaccines are BNT162b2 (mRNA vaccine from Pfizer-BioNTech), mRNA-1273 (mRNA vaccine from Moderna-NIAID), AZD1222 (viral vector from AstraZeneca-University of Oxford), and BBIBP-CorV (inactivated vaccine from Beijing Institute of Biological Products) with 95% (1), 94% (2), 70% (3), and 79% (4) reported efficacy, respectively.

The main concerns about SARS-CoV-2 vaccines are their long-term safety and efficacy, especially in individuals with chronic diseases including cancer. In Iran, BBIBP-CorV has widely been used for in cancer or other chronic diseases. Although BBIBP-CorV (Sinopharm) has been approved in more than 50 countries, there is still limited information on the effectiveness of this vaccine in individuals with pre-existing medical conditions. The results of BBIBP-CorV Phase 3 trial showed positive efficacy and safety data; however, it also revealed that 84.4% of the trial participants were male, 98.4% were under 60 years old, and 100% were healthy (5). This is in contrast with other WHO approved vaccines such as BNT162b2 vaccine (Comirnaty, Pfizer-BioNTech). As for this vaccine, in its Phase 3 trial, 42.2% of the participants were above 55 years old and 20.3% had one or more underlying diseases (1). Some studies and reports from several countries have also indicated that BBIBP-CorV does not produce or produce limited protective antibody in the elderlies (6–8).

Recent review studies indicate the acceptable efficiency of COVID-19 vaccines in cancer patients, although the vaccine efficiency is lower in this group, especially in those with hematological malignancies or those who are actively undergoing chemotherapy (9–14). But most of these studies were done on mRNA vaccines, especially BNT162b2. In Iran, BBIBP-CorV is the most widely used vaccine in the case of malignancies and there are uncertainties about the proper immune response and the possibility of vaccine side effects (13–15).

One study on the immunogenicity and safety of BBIBP-CorV in patients with malignancies, reported the overall seroconversion rate of 86.9% which was lower in older age and chemotherapy receivers and lowest among those with hematologic malignancies with only 61.9%, seroconversion (15).

The Philippine Council for Health Research and Development (PCHRD) also reviewed the studies on the clinical efficacy, effectiveness and safety of BBIBP-CorV in the prevention of SARS-CoV-2 infection as of October 29, 2021. They concluded, due to very low certainty of evidence, it was weakly recommended in adults with comorbidities and older persons (60 years and older) and added, there was insufficient evidence to recommend for or against the use of BBIBP-CorV to prevent COVID-19 infection among immunocompromised population (16).

Based on evidence, COVID-19 causes a significant higher rate of mortality in cancer patients (17–19). Regarding the fact that cancer is also more prevalent in the elderlies, a predictor of poor serologic response to COVID-19 vaccine (11), it is very important to assess the efficacy, durability, and safety of BBIBP-CorV vaccine (the most common vaccine used in Iran) among cancer patients to prevent a possible outbreak of COVID-19 infection in vaccinated but actually unprotected individuals.

Recent studies have indicated that serum neutralizing antibody levels are highly predictive for protection against COVID-19 infection (20–23). Therefore, in the present study we aimed to assess safety and efficacy of BBIBP-CorV in groups of cancer patients using clinical complications of vaccinated individuals after each dose of vaccination. We also made an attempt to assess serum levels of neutralizing antibodies [SARS-CoV anti-spike recombinant receptor binding domain (anti-sRBD), and anti-nucleocapsid (anti-N) IgG].

## Materials and methods

### Participants

In the current case-control study, participants were recruited from among the patients referring to Firoozgar Hospital, affiliated to Iran University of Medical Sciences, Tehran, Iran, within the period of July to October 2021. All patients read and signed informed consent. All the patients were registered to receive BBIBP-CorV vaccine against COVID-19. Two groups of patients with and without history of cancer were considered as case and control groups, respectively. Inclusion criteria were adults (>18 years old) having history of cancer and willingness to receive BBIBP-CorV vaccine selected as cases and adults (≥18 years old) without previous history of cancer and willingness to receive BBIBP-CorV vaccine selected as controls. Exclusion criteria were severe allergic reaction to the vaccine (acute anaphylaxis, angioedema, dyspnea, etc.), severe neurological disorders such as transverse myelitis, Guillain-Barre, and demyelinating diseases, severe uncontrolled chronic diseases, pregnancy, being in the lactation period, recent chemotherapy, and receiving SARS-CoV-2 vaccine other than the BBIBP-CorV or leaving the follow-up meetings.

### Data collection

After obtaining informed consent from all the participants, demographic information, drug history, medical history and co-morbidities, previous history of COVID-19, time to vaccination, and possible side effects of the vaccine were asked according to a questionnaire.

Totally, 2 cc of venous blood sample was obtained from each participant in three points during the study: (1) phase 0: before receiving the vaccine (day 0 or the time of first dose injection), (2) phase I: 28–32 days after the first dose (at the time of the second dose injection), and (3) phase II: 28–32 days after the second dose. The vaccine is in a single-dose (each dose contains 1 cc of water-soluble lyophilized vaccine) for intramuscular injection (the preferred site is deltoid muscle) (10). The recommended schedule is two doses at a recommended interval of 21–28 days. The vaccine in the first and the second injections was of exactly the same type. All the participants in the study received two doses of the vaccine 28 days apart, according to the protocol, to ensure adequate safety (5). Serum levels of antibodies (SARS-CoV anti-sRBD IgG, and anti-N IgG) were assessed accordingly.

Serum levels of SARS-CoV anti-sRBD and anti-N IgG serum levels (Chemobind ELISA Kit, Hayan Pajouh Pars, Tehran, Iran) were measured on days 0, 28–32, and 56–64. The ELISA results are also reported as ratio with the following interpretation: SARS-CoV anti-sRBD: less than 1.1 as negative, and equal or more than 1.1 as positive; SARS-CoV anti-N IgG: less than 1.1 as negative, and equal or more than 1.1 as positive.

Chemobind ELISA kit (Hayan Pajouh Pars, Iran) employs indirect enzyme-linked immune-sorbent technology. The test procedure is as follows: (1) Bring all reagents and specimens to room temperature (18–24°C) before beginning the assay. Swirl gently before use; (2) Add 100 μL of the positive and negative controls into the individual microplate wells according to the pipetting protocol; (3) Add 100 μL of the diluent sample buffer into each well and then add 2 μL of the samples into each well; (4) Incubate the plate at room temperature for 30 min; (5) Wash the strips five times with the working wash solution either manually or with an automatic washer. Leave the wash buffer in each well for 30–60 s per washing cycle, then empty the wells. After washing (manual and automated tests), thoroughly dispose of all liquid from the microplate by tapping it on absorbent paper with the openings facing downward to remove all residual wash buffer; (6) Add 100 μL of Enzyme Conjugate to each well [except blank well]. Mix it gently by swirling the microplate on flat bench and incubate for 30 min at room temperature; (7) Wash the plate five times as described above; (8) Add 100 μL of Chromogen/Substrate to each well. Mix horizontally and incubate at room temperature in the dark for 15 min; (9) Stop the reaction by adding 100 μL of blocking reagent to each well (including the blank) in the same order adopted for the addition of the Chromogen/Substrate solution; (10) After adding the stop solution, read the color developed on the microplate reader at 450 nm. The reading should be done within 30 min from the stop. The reader should be blanked at 450 nm against the blank. Bi-chromatic absorbance measurement with a reference wavelength of 620–650 nm.

#### Diagnostic specificity

The diagnostic specificity is defined as the probability of the assay of scoring negative in the absence of the specific analyte. SARS-CoV-2 infections emerged in December 2019 in Wuhan, China. The expected prevalence values for Iran blood donor panels from before December 2019 therefore amount to 0%. The determined positive results correspond to a specificity of 100%.

#### Diagnostic sensitivity

The diagnostic sensitivity is defined as the probability of the assay of scoring positive in the presence of the specific analyte. A total of 56 patients tested by RT-PCR were evaluated (36 positives, 20 negative). As presented in the following Table, 36 samples were positive for anti-SARS-CoV-2 RBD and 20 samples were negative for anti-SARS-CoV-2 RBD. Thus, sensitivity is 100%.

### Statistical analysis

Descriptive statistics including mean ± standard deviation and median (Interquartile range) were run to express quantitative findings, and frequency (percentage) was used to present qualitative findings. Data were analyzed using the independent sample *t*-test or Mann–Whitney *U*-test, chi-square, or Fisher exact test and repeated measure one-way ANOVA. The equation of variance (sphericity assumption) was assessed running Mauchly’s test of sphericity. Due to the lack of this assumption, the difference between the means at different times was evaluated using the Greenhouse–Geisser correction. Regression analysis was used to compare various dependent and independent variables. The statistical significance level was considered at α: 0.05. All data were analyzed using SPSS, version 22.0, Armonk, NY, USA: IBM Corp. Released 2015.

## Results

### Characteristics of the participants

A total of 152 individuals (67.1% females) with the mean age of 46.71 ± 15.36 years old and mean body mass index (BMI) of 24.96 ± 5.46 kg/m^2^ took part in our study (phase 0). All the participants received the first dose of BBIBP-CorV vaccine and 63 (41.4%) patients reported past history of COVID-19 infection. The participants were divided into two groups: 107 individuals with a history of cancer were selected as cases and 45 with no history of cancer were categorized as controls.

**FIGURE 1:**
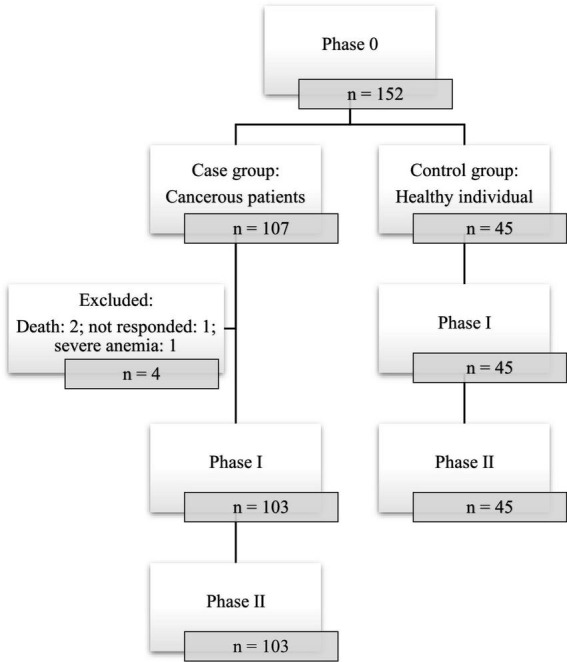
Flow diagram of included individuals in three phases of the study.

Among the cases, solid cancer (87.8%) made up the majority of the cancer types and the most common ones were gynecological (46.7%) and gastrointestinal (31.8%) cancers. Most of the cases were diagnosed at TNM stage II (53.3%) and 15.9% of them were receiving chemotherapy during the study period. In 43% of the cases, it was more than 24 months since cancer diagnosis. Other underlying diseases were present in 38.3% of the cases and the most common diseases were cardiovascular diseases (19.6%) and diabetes (8.4%). Unfortunately, two participants passed out prior to receiving the second dose of the vaccine. Totally, in phase I and II of the study four patients were excluded: two were died due to severe cancer stages, one was none responder, and one had severe anemia which the practitioner avoided sampling (Figure 1).

In the control group, most of the participants (80%) had no underlying diseases and simultaneous diabetes with hypertension (6.7%) was the most frequent one.

The participants in the patient group were significantly older than the controls (52.53 ± 12.86 vs. 32.86 ± 11.54, *p* < 0.001) with significantly higher frequency of other underlying diseases (45.7% vs. 17.7%, *p* = 0.010). Mean anti-sRBD IgG, anti-N IgG serum levels, sex distribution, BMI, past history of COVID-19 infection exercise, and cigarette smoking were not significantly different between the two groups. The descriptive characteristics of the participants are presented in Table 1. Regression analysis of variables including age, gender, smoking, opium usage, exercise, and TNM staging, were not significant (*p*-value > 0.05). However, BMI found significant (*p*-value = 0.015) (OR = 1.149; CI 95%: 1.02–1.28) which could use as a predictive factor in response to vaccination in cancer patients.

**TABLE 1:** Baseline characteristics of the participants (*n* = 152).

	Case (*n* = 107)	Control (*n* = 45)	*p*-value
Age (year) (Mean ± SD)	52.53 ± 12.86	32.86 ± 11.54	<0.001***
BMI (kg/m^2^) (Mean ± SD)	25.27 ± 4.44	23.91 ± 4.05	0.081
Anti-sRBD IgG, GM (CI)	0.86 (0.72–1.03)	1.06 (0.86–1.30)	0.944
Anti-N IgG, GM (CI)	0.74 (0.62–0.88)	0.93 (0.76–1.13)	0.973
Past history of COVID-19 infection	40 (37.4)	23 (51.1)	0.117
**Sex**			
Male	37 (34.6)	13 (28.9)	0.497
Female	70 (65.4)	32 (71.1)	
**Non-oncological comorbidities**			
Diabetes	9 (8.4)	1 (2.2)	0.016*
Autoimmune diseases	1 (0.9)	1 (2.2)	
Cardiovascular diseases	21 (19.6)	1 (2.2)	
Pulmonary diseases	4 (3.7)	1 (2.2)	
Hypothyroid	1 (0.9)	0	
Psychiatric diseases	8 (7.5)	1 (2.2)	
Diabetes and hypertension	5 (4.7)	3 (6.7)	
**Solid cancers**			
Women’s cancers	50 (46.7)	NA	NA
Urological cancers	6 (5.6)	NA	
Thoracic malignancies	1 (0.9)	NA	
Gastrointestinal cancers	34 (31.8)	NA	
Head and neck cancer	3 (2.8)	NA	
**Hematological cancers**			
Lymphoma	7 (6.5)	NA	NA
Nodular sclerosing hodgkin lymphoma	5 (4.7)	NA	
Osteosarcoma	1 (0.9)	NA	
**TNM staging (solid tumors only)**			
I	26 (24.3)	NA	NA
II	57 (53.3)	NA	
III	17 (15.9)	NA	
IV	7 (6.5)	NA	
**Time since cancer diagnosis**			
<3 months	5 (4.7)	NA	NA
3 to <12 months	45 (42.1)	NA	
12–24 months	8 (7.5)	NA	
>24 months	49 (45.8)	NA	
**Cigarette smoking**			
Yes	16 (15)	5 (11)	0.530
No	91 (85)	40 (88.9)	
**Exercise**			
Yes	37 (34.6)	19 (42.2)	0.371
No	70 (65.4)	26 (57.8)	

Data are presented as N (%), unless otherwise specified. Anti-sRBD, anti-spike recombinant receptor binding domain; anti-N, anti-nucleocapsid; BMI, body mass index; CI, confidence interval; GM, geometric mean; NA, not applicable. *p < 0.05; ***p < 0.001. Bolds are significant values.

### Positive anti-sRBD IgG and anti-N IgG at phase 0 (before vaccination)

At Phase 0 of the study or prior to vaccination (day 0 or the time of first dose injection), positive anti-N IgG and anti-sRBD IgG tests were not significantly different between the two groups either in total or sub-analysis according to the past history of COVID-19 infection, sex, BMI or age. Positive anti-sRBD IgG (22.4% of the cases and 13.6% of the controls) and positive anti-N IgG (17.9% of the cases and 13.6% of the controls) were present even with no past history of COVID-19 infection which could be due to the mild and asymptomatic nature of the disease in some people (Tables 2, 3).

**TABLE 2:** Positive anti-sRBD IgG after BBIBP-CorV vaccination.

	Phase 0 (day 0)	*p*-value	Phase I	*p*-value	Phase II	*p*-value
**Total**						
Case	35/107 (32.7%)	0.841	47/103 (45.6%)	0.009**	72/103 (69.9%)	0.002**
Control	14/45 (31.1%)		31/45 (68.9%)		42/45 (93.3%)	
**With past history of COVID-19 infection**						
Case	20/40 (50%)	0.861	29/39 (74.4%)	0.453	35/39 (89.7%)	0.402
Control	11/23 (47.8%)		19/23 (82.6%)		22/23 (95.7%)	
**Without past history of COVID-19 infection**						
Case	15/67 (22.4%)	0.377	18/64 (28.1%)	0.025*	37/64 (57.8%)	0.005**
Control	3/22 (13.6%)		12/22 (54.5%)		20/22 (90.9%)	
**Male**						
Case	9/37 (24.3%)	0.926	10/35 (28.6%)	0.001**	20/35 (57.1%)	0.004**
Control	3/13 (23.1%)		11/13 (84.6%)		13/13 (100.0%)	
**Female**						
Case	26/70 (37.1%)	0.783	37/68 (54.4%)	0.442	52/68 (76.5%)	0.092
Control	11/32 (34.4%)		20/32 (62.5%)		29/32 (90.6%)	
**BMI < 25**						
Case	16/56 (28.6%)	0.603	21/52 (40.4%)	0.269	40/52 (76.9%)	0.032*
Control	6/26 (23.1%)		14/26 (53.8%)		25/26 (96.2%)	
**BMI ≥ 25**						
Case	19/51 (37.3%)	0.719	26/51 (51.0%)	0.003**	32/51 (62.7%)	0.030*
Control	8/19 (42.1%)		17/19 (89.5%)		17/19 (89.5%)	
**Age < 50 years**						
Case	17/45 (37.8%)	0.550	21/44 (47.7%)	0.017*	33/44 (75.0)	0.010*
Control	13/41 (31.7%)		30/41 (73.2%)		39/41 (95.1)	
**Age ≥ 50 years**						
Case	18/62 (29.0%)	0.860	26/59 (44.1%)	0.450	39/59 (66.1)	0.711
Control	1/4 (25.0%)		1/4 (25.0%)		3/4 (75.0)	

Phase 0: before vaccination; phase I: 28–32 days after the first dose; phase II: 28–32 days after the second dose of vaccine. Data are presented as N (%). anti-sRBD, anti-spike recombinant receptor binding domain. *p < 0.05; **p < 0.01. Bolds are significant values.

**TABLE 3:** Positive anti-N IgG after BBIBP-CorV vaccination.

	Phase 0 (day 0)	*p*-value	Phase I	*p*-value	Phase II	*p*-value
**Total**						
Case	31/107 (29%)	0.792	43/103 (41.7%)	0.005**	51/103 (49.5%)	<0.001***
Control	14/45 (31.1%)		30/45 (66.7%)		38/45 (84.4%)	
**With past history of COVID-19 infection**						
Case	19/40 (47.5%)	0.981	28/39 (71.8%)	0.160	28/39 (71.8%)	0.068
Control	11/23 (47.8%)		20/23 (87%)		21/23 (91.3%)	
**Without past history of COVID-19 infection**						
Case	12/67 (17.9%)	0.642	15/64 (23.4)	0.050	23/64 (35.9%)	0.001**
Control	3/22 (13.6%)		10/22 (45.5)		17/22 (77.3%)	
**Male**						
Case	10/37 (27%)	0.433	9/35 (25.7%)	0.001**	13/35 (37.1%)	<0.001***
Control	5/13 (38.5%)		10/13 (76.9%)		13/13 (100.0%)	
**Female**						
Case	21/70 (30%)	0.840	34/68 (50%)	0.241	38/68 (55.9%)	0.032*
Control	9/32 (28.1%)		20/32 (62.5%)		25/32 (78.1%)	
**BMI < 25**						
Case	12/56 (21.4%)	0.860	20/52 (38.5%)	0.540	24/52 (46.2%)	0.001**
Control	6/26 (23.1%)		16/26 (61.5%)		22/26 (84.6%)	
**BMI ≥ 25**						
Case	19/51 (37.3%)	0.712	23/51 (45.1%)	0.033*	27/51 (52.9%)	0.017*
Control	8/19 (42.1%)		14/19 (73.7%)		16/19 (84.2%)	
**Age < 50 years**						
Case	10/45 (22.2%)	0.452	20/44 (45.5%)	0.059	27/44 (61.4%)	0.013*
Control	12/41 (29.3%)		27/41 (65.9%)		35/41 (85.4%)	
**Age ≥ 50 years**						
Case	21/62 (33.9%)	0.519	23/59 (39.0%)	0.159	24/59 (40.7%)	0.171
Control	2/4 (50.0%)		3/4 (75.0%)		3/4 (75.0%)	

Phase 0: before vaccination; phase I: 28–32 days after the first dose; phase II: 28–32 days after the second dose of vaccine. Data are presented as n/N (%). Anti-N, anti-nucleocapsid; BMI, body mass index. *p < 0.05; **p < 0.01; ***p < 0.001. Bolds are significant values.

### Positive anti-sRBD IgG and anti-N IgG at phase I and side effects

As for Phase I, 28–32 days after the first dose, positive anti-sRBD IgG test was significantly lower among the cases (45.6% vs. 68.9%, *p* = 0.009) in total analysis. In sub-analysis, positive anti-sRBD IgG test was significantly lower in cases with no history of COVID-19 infection (28.1% vs. 54.5%, *p* = 0.025), males (28.6% vs. 84.6%, *p* = 0.001), those with BMI ≥ 25 (51.0% vs. 89.5%, *p* = 0.003), and those younger than 50 years of age (47.7% vs. 73.2%, *p* = 0.017) compared with those of their controls (Table 2).

Positive anti-N IgG test was significantly lower among the cases (41.7% vs. 66.7%, *p* = 0.005) in total analysis. In sub-analysis, positive anti-N IgG test was found to be significantly lower in male cases (25.7% vs. 76.9%, *p* = 0.001) or those with BMI ≥ 25 (45.1% vs. 73.7%, *p* = 0.033) compared to their control counterparts; however, analyses of past history of COVID-19 infection and age did not reveal any significant effect (Table 3).

Moreover, presence of side effects was not observed to be significantly different between the case and control groups. Furthermore, 80.4% of the cases and 77.8% of the controls had no side effect and the most common side effects were pain and redness at the injection site, headache, myalgia, and fever in the two groups (Table 4).

**TABLE 4:** Side effects of BBIBP-CorV vaccination after the first and the second dose.

Side effects after the first dose								
Groups	Fever	Headache	Nausea	Myalgia	Pain and redness at the injection site	Combination of the side effects	No side effects	*p*-value
Case (*n* = 103)	3 (2.9)	4 (3.8)	0	4 (3.8)	6 (5.8)	4 (3.8)	86 (83.4)	0.371
Control (*n* = 45)	1 (2.2)	4 (8.9)	0	0	1 (2.2)	4 (8.9)	35 (77.8)	
**Side effects after the second dose**								
Case (*n* = 103)	3 (2.9)	3 (2.9)	1 (0.9)	5 (4.8)	5 (4.8)	2 (1.9)	88 (85.4)	0.990
Control (*n* = 45)	1 (2.2)	1 (2.2)	0	1 (2.2)	2 (4.4)	1 (2.2)	39 (86.7)	

Data are presented as N (%).

### Positive anti-sRBD IgG and anti-N IgG at phase II and side effects

It was found that 28–32 days after the second dose (Phase II), positive anti-sRBD IgG test was significantly lower among the cases (69.9% vs. 93.3%, *p* = 0.002). In sub-analysis, positive anti-sRBD IgG test was significantly lower among the cases without past history of COVID-19 (57.8% vs. 90.9%, *p* = 0.005), in males (57.1% vs. 100%, *p* = 0.004), in both groups of BMIs (BMI < 25: 76.9% vs. 96.2%, *p* = 0.032; BMI ≥ 25: 62.7% vs. 89.5%, *p* = 0.030) and in those younger than 50 years old (75% vs. 95.1%, *p* = 0.010) compared to their peers in the control group (Table 2).

Positive anti-N IgG test was significantly lower among the cases (49.5% vs. 84.4%, *p* < 0.001) in total analysis. In sub-analysis, positive anti-N IgG test was found to be significantly lower among the cases without past history of COVID-19 infection (35.9% vs. 77.3%, *p* = 0.001), in both males (37.1% vs. 100%, *p* < 0.001) and females (55.9% vs. 78.1%, *p* = 0.032), in both groups of BMIs (BMI < 25: 46.2% vs. 84.6%, *p* = 0.001; BMI ≥ 25: 52.9% vs. 84.2%, *p* = 0.017), and in those younger than 50 years old (61.4% vs. 85.4%, *p* = 0.013) compared to their peers in the control group (Table 3).

Presence of side effects were not significantly different between the case and control groups. In fact, 82.2% of the cases and 86.7% of the controls had no side effect. The most common side effects were pain and redness at the injection site, headache, myalgia, and fever in both groups (Table 4).

### Positive anti-sRBD IgG after the second dose in subgroups of cancer patients

Positive anti-sRBD and anti-N tests were not significantly different between solid and hematological cancers 28–32 days after the first and the second doses. No significant difference was revealed between different 4 TNM stages either (Table 5). However, positive anti-sRBD IgG results were significantly different between categories of time since the last chemotherapy session. The lowest positive anti-sRBD IgG test was among the patients who were simultaneously receiving chemotherapy (Table 6).

**TABLE 5:** Positive anti-sRBD IgG after second dose BBIBP-CorV vaccination among different classifications in cancer patients.

	Positive anti-sRBD IgG at phase II	*p*-value	Positive anti-N IgG at phase II	*p*-value
**Type of cancer**				
Solid cancers	66/91 (72.5)	0.118	47/91 (51.6)	0.236
Hematological cancers	6/12 (50.0)		4/12 (33.3)	
**TNM staging**				
I	18/25 (72.0)	0.161	13/25 (52)	0.476
II	43/57 (75.4)		29/57 (50.9)	
III	8/17 (47.1)		6/17 (35.3)	
IV	3/4 (75)		3/4 (75)	

Phase II: 28–32 days after the second dose of vaccine. Data are presented as n/N (%). Anti-sRBD, anti-spike recombinant receptor binding domain.

**TABLE 6:** Results of anti-N and anti-S antibodies assessment in different phases of the study based on cancer treatment strategy.

	Therapy time^	Cases	N0 Mean ± SD	N1 Mean ± SD	N2 Mean ± SD	*p*-value*	S0 Mean ± SD	S1 Mean ± SD	S2 Mean ± SD	*p*-value*
Chemotherapy	Total	54	1.18 ± 1.30	1.31 ± 1.11	2.49 ± 2.45	**0.041**	1.29 ± 1.53	1.73 ± 1.89	2.57 ± 2.10	**0.001**
	Before	40	1.25 ± 1.38	1.40 ± 1.17	3.06 ± 2.60	**0.010**	1.35 ± 1.48	2.00 ± 2.10	2.94 ± 2.12	0.081
	Simultaneous	14	0.97 ± 1.01	1.03 ± 0.88	0.85 ± 0.50		1.11 ± 1.70	0.93 ± 0.64	1.50 ± 1.72	
Chemo-Radiotherapy	Total	14	0.75 ± 0.53	1.47 ± 1.27	3.65 ± 3.23	**0.001**	1.27 ± 1.61	1.95 ± 1.47	2.70 ± 1.41	**0.005**
	Before	14	0.75 ± 0.53	1.47 ± 1.27	3.65 ± 3.23	–	1.27 ± 1.61	1.95 ± 1.47	2.70 ± 1.41	–
	Simultaneous	0	–	–	–		–	–	–	
Endocrine	Total	10	1.51 ± 1.61	0.87 ± 0.81	1.70 ± 1.81	0.188	1.66 ± 2.15	1.52 ± 1.59	2.70 ± 2.19	**0.004**
	Before	0	–	–	–	–	–	–	–	–
	Simultaneous	10	1.51 ± 1.61	0.87 ± 0.81	1.70 ± 1.81		1.66 ± 2.15	1.52 ± 1.59	2.70 ± 2.19	
Other therapies^&^	Total	16	0.74 ± 0.72	0.85 ± 0.75	1.83 ± 2.06	0.142	0.91 ± 1.04	1.40 ± 1.48	1.83 ± 1.57	**0.009**
	Before	2	1.86 ± 1.54	1.96 ± 1.75	4.42 ± 5.33	**0.020**	0.65 ± 0.03	2.30 ± 2.26	2.50 ± 2.63	0.228
	Simultaneous	14	0.58 ± 0.44	0.69 ± 0.45	1.46 ± 1.23		0.94 ± 1.11	1.28 ± 1.65	1.73 ± 1.49	
No cancer therapy	Total	13	1.86 ± 2.27	2.12 ± 1.60	2.04 ± 2.53	**0.046**	2.55 ± 2.36	2.97 ± 1.91	3.14 ± 2.04	0.600
Total	Total	107	1.07 ± 1.17	1.20 ± 1.06	2.47 ± 2.50	**0.001**	1.26 ± 1.53	1.68 ± 1.78	2.47 ± 1.93	**0.001**
	Before	56	1.14 ± 1.23	1.44 ± 1.19	3.26 ± 2.82	**0.001**	1.31 ± 1.48	2.00 ± 1.98	2.86 ± 1.94	**0.025**
	Simultaneous	38	0.96 ± 1.09	0.86 ± 0.72	1.31 ± 1.25		1.20 ± 1.63	1.22 ± 1.34	1.91 ± 1.80	

^&^Other therapies: chemo-immunotherapy (*n* = 4); radiotherapy (*n* = 3); immuno-radiotherapy (*n* = 2); chemo-radio-endocrine therapy (*n* = 2); chemo-TKI and target therapy (*n* = 1); chemo-endocrine therapy (*n* = 1); radio-endocrine therapy (*n* = 1); chemo-radio-immunotherapy (*n* = 1); immunotherapy (*n* = 1).

^Time of cancer treatment means the cases finished their cancer treatments before vaccination and cases obtained treatment during vaccination time simultaneously.

*One-way ANOVA repeated measures. Bolds are significant values.

### Comparison of anti-sRBD IgG and anti-N IgG serum levels following the first and the second doses of vaccination

Anti-sRBD IgG and anti-N IgG serum levels increased significantly 28–32 days after the first and the second doses both in total analysis and in each group (cancer patients and the controls) separately (*p* < 0.001). Both anti-sRBD IgG and anti-N IgG serum levels were significantly higher in the control group (*p* = 0.035 and *p* = 0.020, respectively).

**FIGURE 2:**
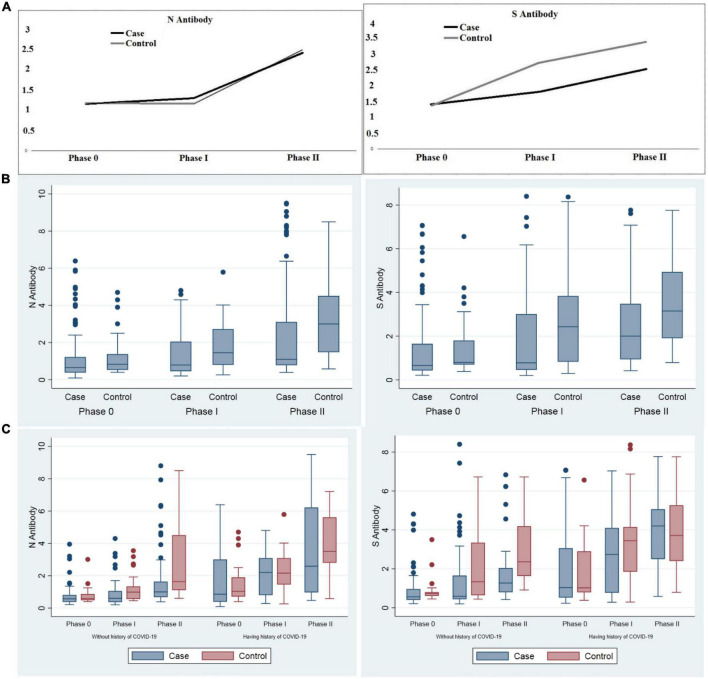
Comparison of anti-sRBD IgG and anti-N IgG serum levels before vaccination, 28–32 days after the first and the second dose among cancer patients and the control group. **(A)** Trend of anti-sRBD IgG and anti-N IgG **(B)** total anti-sRBD IgG and anti-N IgG against SARS CoV2 in each phases of the study; **(C)** sub-group analysis by past history of COVID-19 infection. Phase 0: before vaccination; phase I: 28–32 days after the first dose; phase II: 28–32 days after the second dose of vaccine. S0, S1, S2: anti-spike recombinant receptor binding domain IgG before vaccination, 28–32 days after the first and the second dose respectively; N0, N1, N2: anti-nucleocapsid IgG before vaccination, 28–32 days after the first and the second dose respectively.

The increase in serum anti-sRBD IgG during the three phases was significantly more in the control group in comparison with that of the cancer patients (*p* = 0.002), yet serum anti-N IgG changes were not observed to be significantly different between the two groups (*p* = 0.621) (Table 7; Figure 2).

**TABLE 7:** Comparison of anti-sRBD IgG and anti-N IgG serum levels before and at two intervals after BBIBP-CorV vaccination.

	Groups	Mean	Std. deviation	Between-groups	Time × group	Time
**N antibody**						
N0	Case	1.16	1.35	0.020*	0.621	**<0.001** ***
	Control	1.18	1.02			
	Total	1.17	1.26			
N1	Case	1.31	1.16			
	Control	1.83	1.24			
	Total	1.47	1.21			
N2	Case	2.42	2.49			
	Control	3.32	2.15			
	Total	2.69	2.42			
**S antibody**						
S0	Case	1.41	1.69	0.035*	0.002**	**<0.001** ***
	Control	1.39	1.26			
	Total	1.40	1.56			
S1	Case	1.83	1.83			
	Control	2.74	2.07			
	Total	2.11	1.95			
S2	Case	2.55	1.95			
	Control	3.42	1.79			
	Total	2.82	1.93			

S0, S1, S2: anti-spike recombinant receptor binding domain IgG before vaccination, 28–32 days after the first and the second dose respectively; N0, N1, N2: anti-nucleocapsid IgG before vaccination, 28–32 days after the first and the second dose respectively. *p < 0.05; **p < 0.01; ***p < 0.001. Bolds are significant values.

### Comparison of anti-sRBD IgG and anti-N IgG serum levels in different phases of the study based on cancer treatment strategy

**FIGURE 3:**
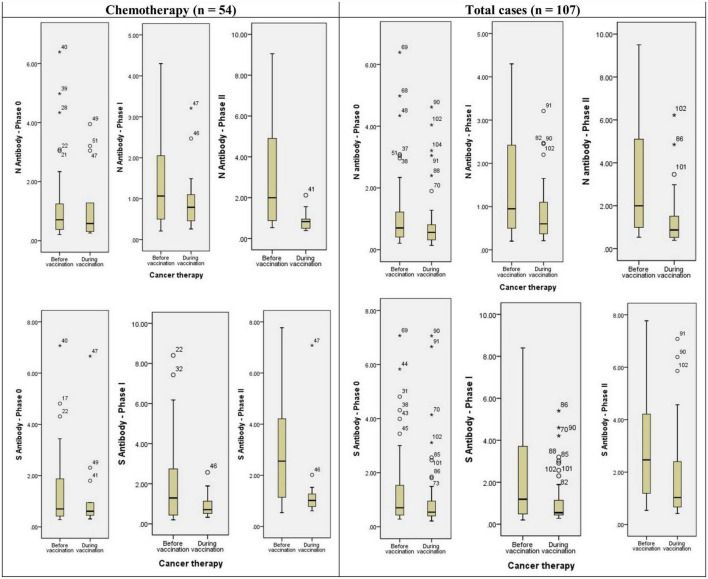
Diagram of anti-N and anti-S antibodies assessment in different phases of the study based on cancer treatment strategy. * or ° represents sample code.

Anti-sRBD IgG and anti-N IgG serum levels increased significantly 28–32 days after the first and the second doses both in the general analysis of different cancer treatment methods (anti-sRBD IgG and anti-N IgG: *p* = 0.001), and in the subgroup analysis based on the time of cancer treatment [before or simultaneous with vaccine injection (anti-sRBD IgG: *p* = 0.025 and anti-N IgG: *p* = 0.001)] (Table 6; Figure 3).

Anti-N IgG serum level increased significantly in patients receiving chemotherapy both before or simultaneous with vaccine injection (both, *p* = 0.041). There were 14 patients who had received chemo-radiotherapy before injection and they also had a significant increase in anti-N IgG serum level (*p* = 0.001). Patients receiving endocrine therapy didn’t show a significant increase in anti-N IgG serum level and they were all receiving the treatment simultaneously with the vaccine injection. Anti-N IgG serum level increase was also significant in patients who used other treatment methods, both before or simultaneous with the vaccine injection (both, *p* = 0.020) but not in total analysis. Those patients who didn’t receive any kind of cancer treatment also had a significantly increased anti-N IgG serum level (*p* = 0.04) (Table 6; Figure 3).

Anti-s RBD IgG serum level increased significantly in patients receiving chemotherapy in total analysis (*p* = 0.001), but not in its subgroups (before or simultaneous with vaccine injection). The patients who had received chemo-radiotherapy before injection (*p* = 0.005) and those receiving endocrine therapy simultaneously with the vaccine injection (*p* = 0.004) also had a significant increase in anti-s RBD IgG serum level. Anti-s RBD IgG serum level increase was also significant in patients who used other treatment methods in total analysis (*p* = 0.009), but not in its subgroups. The patients with no kind of cancer treatment did not show a significant increase in anti-s RBD IgG serum level (*p* = 0.046) (Table 6; Figure 3).

## Discussion

It has been documented that appropriately evaluated antibody titers are in close association with disease susceptibility, especially anti-sRBD which has a key role in the binding and cellular entry of the SARS-CoV-2 virus and has been used in the phases I and II studies of the RNA-based vaccines (20–23). In this case-control study, SARS-CoV-2 virus neutralizing antibody titer was used as a measure of diseases protection in a group of cancer patients vaccinated with BBIBP-CorV vaccine. Based on the results of this study, Anti-sRBD IgG and anti-N IgG serum levels increased significantly 28–32 days after the first and the second dose in both cancer patients and the controls but their serum levels were significantly lower among cancer patients. Accordingly, the frequency of positive antibody response (ratio equal to or more than 1.1) for both anti-sRBD and anti-N IgG were significantly lower among cancer patients compared to those of the controls. This lower frequency of positive antibody response among cancer patients was especially observed in participants without previous history of COVID-19 infection, males, and individuals younger than 50.

The effect of a history of COVID-19 infection on the results might be due to the higher antibody response in participants with the positive history. Buonfrate et al. (24) reported that individuals with past COVID-19 infection had a strong antibody response even after one single dose. Ariamanesh et al. (15) also reported a more robust response of the SARS-CoV2 Spike protein, in cancer patients with a history of COVID-19 infection compared to those without such history. So, higher antibody response in patients with a history of COVID-19 infection might have reduced the difference in antibody response between cancer patients and controls.

In the current study, sex was also found to have a significant effect on antibody response especially on anti-sRBD IgG. Anti-sRBD IgG response was significantly lower in male cancer patients compared to their peers in the control group 28–32 days after the second dose of BBIBP-CorV, while no significant difference was observed among females. Vassilaki (25) also reported that antibody titers were slightly higher in the female population following the second dose of BNT162b2 vaccine. Javadinia et al. (26) also report that seropositivity against COVID-19 is higher in females. This effect might be due to the enhancing effect of female hormones on the immune system (27). But in contrast to these results, sex had little association with antibody titers after BBIBP-CorV in the two studies by Ferenci et al. (6) and Jabal et al. (28).

Age was another affecting factor on antibody response in this analysis. In participants <50 years old, antibody response (both anti-sRBD and anti-N IgG) was significantly lower among cancer patients compared to those in the control group while no significant difference was observed in participants ≥50 years. However, we had not enough controls upper 50 years to conclude better. Age matching between case and control group may contribute to a selection bias in the present study and should interpret by more caution.

According to the findings by Ferenci et al. (6), a significant association exists between age and antibody levels, with significantly lower antibody titers in older individuals (14, 29). Therefore, lower anti-body response among the older control group might have decreased the difference of anti-body response between cancer patients and those in the control group.

In the present study, 66.1% of cancer patients and 75% of controls who were ≥50 years old had anti-sRBD response after full vaccination with BBIBP-CorV. This finding is similar to those by Ferenci et al. (6) who reported the antibody response of 75% at 60 years of age after full vaccination with BBIBP-CorV. But this result for Pfizer-BioNTech vaccine is different. Although Pfizer-BioNTech vaccine too showed a negative association between age and antibody titer, no subject had negative antibody test (i.e., a titer below 1) and its probability was estimated to be less than 10% even in the oldest age group (6). Richards et al. (30) also reported lower antibody titers in older individuals after full vaccination with Pfizer-BioNTech but no subject had negative antibody test (i.e., a titer below 1), either. These results indicate that Pfizer-BioNTech vaccine might be a better choice in cancer patients who are usually in their older ages.

Body mass index might be another factor which affects the antibody response after vaccination including the fact that, although in the present study, antibody response (both anti-sRBD and anti-N IgG) were significantly lower among the cancer patients in both BMI groups (BMI ≥ 25 and BMI < 25), lower anti-sRBD IgG response of cancer patients was more considerable in the group with BMI ≥ 25. Some studies indicate that obesity may decrease vaccine-induced immunogenicity and higher BMI has been associated with lower titers of antibodies against SARS-CoV-2, especially in men (31, 32).

In the present study, solid cancers had a better antibody response compared to hematologic cancers although the difference was not significant and their population was not matched (91 cases vs. 12 cases). Other studies have also reported better antibody response in solid cancers. For instance, studies by Monin et al. (33) and Massarweh et al. (34) both indicated antibody responses in most of the patients with solid cancers, but antibody response has been weaker in patients with hematologic cancers (15, 35). A systematic review by Javadinia et al. (14), also indicate a higher seroconversion rate in solid malignancies (88%) compared to hematologic malignancies (70%). Yet, the point is that, antibody response after full vaccination with BBIBP-CorV in our study was 71.9% in solid cancers and 50% in hematologic cancers, which is much lower compared to those reported by Monin et al. (33), who revealed the antibody response of 95% in solid cancers and 60% in hematologic cancers after full BNT162b2 (Pfizer-BioNTech) vaccination. It seems that, compared to BBIBP-CorV, BNT162b2 (Pfizer-BioNTech) vaccine is more efficient in protecting cancer patients, even the hematologic ones, against COVID-19. The antibody response of solid cancers in the present study was also lower than the reported antibody response of 85.7% in a group of breast cancer patients receiving BBIBP-CorV (36). This might be due to the presence of different solid cancers in the current study instead of one single type. However, hematological cancer patients commonly received anti-CD20 monoclonal antibody drugs which exhausts B-cells and they have reduced antibody production and consequently, vaccine antibody response affected negatively.

According to the findings of this study only simultaneous endocrine treatment may inhibit significant increase in anti-N IgG serum level. Other types of cancer treatment and the time of their administration were not effective on its level. Anti-sRBD serum level was not either affected by the type and the administration time of cancer treatment. This is in contrast with previous studies indicating that impaired serological response was mostly observed in active chemotherapy or highly immunosuppressive therapies (29, 37). The type of vaccine may have intervened in these discrepancies, as the vaccines assessed in previous studies were mRNA based, while in this study a whole inactivated virus vaccine was used.

No life-threatening side effects were observed during the current study, and like previous reports, the most common side effect were pain and redness at the injection site as well as headache.

Altogether, in the present study, antibody response (both anti-sRBD and anti-N IgG) were significantly lower among cancer patients, as compared with the controls, after full vaccination with BBIBP-CorV. Males, individuals with BMI ≥ 25, and those without previous history of COVID-19 infection as well as hematologic cancers and those with simultaneous endocrine treatment were more likely to have lower antibody response. The third dose vaccination may a good choice to boost their immunity as well.

More than 50 countries have approved BBIBP-CorV vaccine. Millions of people have been vaccinated or are going to be vaccinated with this vaccine in near future. Therefore, it is utterly vital to have evidence on safety and effectiveness of this vaccine particularly among those with possible weaker antibody response, such as patients with malignancies, older individuals, as well as overweight or obese adults. This is very important to prevent an outbreak of COVID-19 infection in those who are apparently vaccinated but are actually unprotected. Moreover, there is a study calculate the highest antibody response in cancer patients by BNT162b2 vaccine compared with other vaccines (9).

To the best of our knowledge, this is the first case-control study on BBIBP-CorV disease protection capability in cancer. Nevertheless, this study has some limitations that should be taken into consideration. Although we tried to separate subjects with and without past history of COVID-19 infection, there might have been asymptomatic patients who have negatively affected the results as an intervening variable. In this regard, asymptomatic patients could have elevated antibodies (26) which they put into the group of without history of COVID-19 and may interfere with our group classification. Also, age matching between cases and controls was another limitation of our study that may impact on our conclusion about rate of antibody response and comorbidity status in this group compared with cases. Thus, the age matching bias may have impact on our response to vaccination (52.53 ± 12.86 vs. 32.86 ± 11.54). Actually, using a written consent, age more than 18 years, excluding some cases with higher grade of malignancy, those susceptible to sampling, limited study duration, restricted number of referred cases to our center in order to using vaccination schedule in every available other center, using one single center for the study, our sample size declined compared to some other studies which recommended greater population for further studies.

## Conclusion

Full vaccination against COVID-19 could reduce morbidity and related mortality not only for general population but even for immunocompromised population such as under treatment cancer patients. The BBIBP-CorV vaccine has minor side effect and recommend for massive usage in various populations. Also, by our study we found antibody response was significantly lower among cancer patients compared to that in the controls after full vaccination with BBIBP-CorV. Lower antibody response may relate to male sex, those with BMI ≥ 25, individuals with no history of COVID-19 infection, participants with hematologic cancers, or simultaneous endocrine treatment. Older individuals had lower anti-body response, which can indicate extra doses of the vaccine. Pain, redness at the injection site, and headache were the most common side effects of BBIBP-CorV vaccine. More thorough studies with greater sample sizes and reducing selection bias strictly are recommended to obtain more comprehensive results for future pandemics.

## Data availability statement

The data supporting the conclusions of this article will be made available by the corresponding author by the reasonable request.

## Ethics statement

The studies involving human participants were reviewed and approved by Iran University of Medical Sciences, Tehran, Iran (code: IR.IUMS.FMD.REC.1400.250). The patients/participants provided their written informed consent to participate in this study.

## Author contributions

SJ, MJ, FZ, and AB contributed to the patients sampling and data collection. AG, SA, FS, and SJ contributed to the laboratory tests analysis and interpretation the results. YA and MK contributed to the statistical analysis. BA and FS contributed to the writing the manuscript draft. MK, FS, and BA contributed to the critical revision and finalize the manuscript revisions. All authors read and approved the final manuscript.
